# EMT transcription factor ZEB1 alters the epigenetic landscape of colorectal cancer cells

**DOI:** 10.1038/s41419-020-2340-4

**Published:** 2020-02-24

**Authors:** Pablo Lindner, Sushmita Paul, Markus Eckstein, Chuanpit Hampel, Julienne K. Muenzner, Katharina Erlenbach-Wuensch, Husayn P. Ahmed, Vijayalakshmi Mahadevan, Thomas Brabletz, Arndt Hartmann, Julio Vera, Regine Schneider-Stock

**Affiliations:** 1 Institute of Pathology, Universitätsklinikum Erlangen, Friedrich-Alexander University of Erlangen-Nuremberg, Bavaria, Germany; 2 Experimental Tumorpathology, Universitätsklinikum Erlangen, Friedrich-Alexander University of Erlangen-Nuremberg, Bavaria, Germany; 3 Laboratory of Systems Tumor Immunology, Department of Dermatology, Universitätsklinikum Erlangen, Friedrich-Alexander University of Erlangen-Nuremberg, Bavaria, Germany; 4 Institute of Bioinformatics and Applied Biotechnology (IBAB), Bangalore, India; 5 Experimental Medicine I, Nikolaus-Fiebiger-Center for Molecular Medicine, Comprehensice Cancer Center, Universitätsklinikum Erlangen, Friedrich-Alexander University of Erlangen-Nuremberg, Erlangen, Germany

**Keywords:** Gene silencing, Colon cancer

## Abstract

Epigenetic deregulation remarkably triggers mechanisms associated with tumor aggressiveness like epithelial–mesenchymal transition (EMT). Since EMT is a highly complex, but also reversible event, epigenetic processes such as DNA methylation or chromatin alterations must be involved in its regulation. It was recently described that loss of the cell cycle regulator p21 was associated with a gain in EMT characteristics and an upregulation of the master EMT transcription factor ZEB1. In this study, in silico analysis was performed in combination with different in vitro and in vivo techniques to identify and verify novel epigenetic targets of ZEB1, and to proof the direct transcriptional regulation of SETD1B by ZEB1. The chorioallantoic-membrane assay served as an in vivo model to analyze the ZEB1/SETD1B interaction. Bioinformatical analysis of CRC patient data was used to examine the ZEB1/SETD1B network under clinical conditions and the ZEB1/SETD1B network was modeled under physiological and pathological conditions. Thus, we identified a self-reinforcing loop for ZEB1 expression and found that the SETD1B associated active chromatin mark H3K4me3 was enriched at the ZEB1 promoter in EMT cells. Moreover, clinical evaluation of CRC patient data showed that the simultaneous high expression of ZEB1 and SETD1B was correlated with the worst prognosis. Here we report that the expression of chromatin modifiers is remarkably dysregulated in EMT cells. SETD1B was identified as a new ZEB1 target in vitro and in vivo. Our study demonstrates a novel example of an activator role of ZEB1 for the epigenetic landscape in colorectal tumor cells.

## Introduction

Activation of a partial epithelial–mesenchymal transition (EMT) is a crucial cellular process for invasion and metastasis^1^. During EMT, epithelial cells lose cell–cell junctions, reorganize the cytoskeleton, gain a spindle-shaped morphology and increase cell motility. EMT in epithelial cancer can be found in invading cells mostly at the tumor invasion front with a loss of E-cadherin and sometimes an upregulation of Vimentin reflecting their high differentiation plasticity^2^. Such cells likely represent the most aggressive clones in a tumor that are responsible for malignant progression and metastasis.

A small group of pleiotropic transcription factors is responsible for the regulation of the EMT program. The double zinc finger and homeodomain factor ZEB1 is one of the most potent EMT-activators^3,4^ that is associated with aggressive behavior, metastasis, treatment resistance, and poor prognosis in different cell types^5,6^. It has been shown that ZEB1 represses the expression of epithelial genes and that this ability is an important feature for metastasis^6–9^. Since EMT is a highly complex, but reversible phenomenon, it seems to be logic that epigenetic mechanisms such as DNA methylation or chromatin alterations must be involved in EMT regulation.

So far, there are only few reports on how ZEB1 is associated with chromatin regulation. In fact, ZEB1 inhibits gene expression not only by direct DNA binding, but also by forming a repressor complex with Sirt1, a class III histone deacetylase (HDAC), to bind the promoter of E-cadherin, leading to E-cadherin expression silencing and the induction of EMT in prostate and pancreatic cancer cells^10,11^. ZEB1 might recruit the histone deacetylase HDAC1 or the methyltransferase DNMT1 to the E-cadherin promoter to repress its transcription and maintain its hypermethylation status, respectively^11^. Nevertheless, if ZEB1 can directly regulate the expression of chromatin modification enzymes and if these chromatin modifiers are essential for the progression or inhibition of EMT has remained widely elusive.

Recently, it has been reported that the cyclin-dependent kinase inhibitor p21 (p21^Cip1/WAF1^) prevents EMT in tumors through inhibiting the expression and activity of the EMT transcription factor ZEB1^12^. Here, p21 knockout cells (p21–/–) have been described to undergo a partial EMT showing a phenotypic heterogeneity and cellular plasticity with mesenchymal and epithelial characteristics. Yet, while EMT has been largely studied by examining “pure” epithelial or mesenchymal states, transient phenotypes still remain poorly understood, mainly because they are quite difficult to capture in vivo^13^. Thus, the p21 knockout model is a suitable system to identify novel players of EMT with relevance in cancer progression and therapy.

In this study, we report on an epigenetically regulated, self-reinforcing loop for ZEB1-mediated EMT regulation, when the cell cycle inhibitor p21 is lost. For the first time, we describe a ZEB1-dependent dysregulation of chromatin modifiers in vitro and in vivo in the chicken CAM model. Furthermore, we show that there seems to be an active role of ZEB1 in shaping the epigenetic landscape to realize the EMT associated gene expression signature.

## Materials and methods

### Cell culture

For details, please see the Supplementary methods. The following cell lines were used: HCT116 (HCT WT, HCT p21–/–, HCT p53–/–) and DLD-1 (DLD-1 WT, DLD-1 p21–/–). Cells were cultured at 37 °C with 5% CO_2_. Mycoplasma free status was verified. All cell lines were authenticated using multiplex cell authentication by multiplexion (Heidelberg, Germany). Phase-contrast images were acquired using Leica instruments (Leica, Wetzlar, Germany). Images were edited with Adobe PhotoShop CS5 (Adobe Systems Inc., DW, USA) and ImageJ software (National Institute of Health, USA).

### Collection of cell pellets

A detailed depiction is placed in the Supplementary methods. Cells were washed, scraped off the culture dish and transferred into sterile tubes. Cells were centrifuged and divided for protein (~60%), DNA (~10%) and RNA (~30%) preparation. Cell pellets were frozen in liquid N_2_ and stored at −80 °C.

### Western blotting

A detailed protocol with a list of used antibodies can be found in the Supplements. Briefly, protein lysates were separated by SDS-PAGE and blotted on nitrocellulose membranes overnight. Protein bands were detected, and images were processed using Adobe PhotoShop CS5 (Adobe Systems Inc., DW, USA) and ImageJ software (National Institute of Health, USA). Ratios were calculated against the house keeper GAPDH.

### RNA expression analysis

Detailed descriptions and protocols can be found in the Supplements. Briefly, from total RNA a reverse transcription was executed, and cDNA amplification was accomplished with gene-specific primers and SYBR^®^ Green based kits using the CFX96^TM^ Real-Time System (Bio-Rad, Munich, Germany). A list of primers for RT-qPCR and gene lists for RT² Profiler PCR Arrays (Qiagen, Hilden, Germany) can be found in the Supplementary information. Expression values were normalized to human B2M or GAPDH expression and given as relative fold expression compared to respective controls.

### ZEB1 and SETD1B knock-down—siRNA transfection

A detailed description and the used siRNA sequences can be found in the Supplements. In short, cells were transfected and collected as described before at 24 and 48 h after transfection start. Transfection experiments were performed as recently explained^14^.

### ZEB1 overexpression—plasmid transfection

A detailed description can be found in the Supplements. Briefly, cells were transfected for 6 h and collected at 24 and 48 h. Transfection experiments were performed as recently explained^14^.

### Immunohistochemistry

A set of detailed protocols for antibody staining and antibody dilutions can be found in the Supplements. Briefly, FFPE sections were deparaffinized, rehydrated and stained for HE or with different antibodies. Bright field images were taken using Olympus instruments (Olympus Corporation, Shinjuku, Japan).

### Immunohistochemistry—assessment of mitoses

A detailed description can be found in the Supplementary. Briefly, HE stained FFPE sections were digitized and five high power fields (HPF) per slide were analyzed and compared between HCT WT and HCT p21–/– cells.

### Co-immunoprecipitation

Co-Immunoprecipitation was performed according to the manufacturer’s protocol and the following kit was used: Dynabeads^®^ Protein G Immunoprecipitation Kit (Thermo Fischer, Waltham, USA; #10007D). Briefly, cell lysates (1000 µg per sample) were incubated with the primary antibodies over night at 4 °C with rotation. Following primary antibody was used: SETD1B (Abcam, Cambridge, UK; #ab113984; 10 µg per sample). The next day, the targets were eluted and stored for further use or directly analyzed by western blotting as recently described.

### Proximity ligation assay (PLA)

The PLA was performed according to the manufacturer’s manual and the following reagents were used: Duolink^®^ In Situ Detection Reagents Red (Sigma-Aldrich, Darmstadt, Germany; #DUO92008-100RXN), Duolink^®^ In Situ PLA^®^ Probe Anti-Mouse PLUS (Sigma-Aldrich, Darmstadt, Germany; #DUO92001-100RXN) and Duolink^®^ In Situ PLA^®^ Probe Anti-Rabbit MINUS (Sigma-Aldrich, Darmstadt, Germany; #DUO92005-100RXN). In short, the cells were co-stained over night at 4 °C using the following antibodies: SETD1B (Abcam, Cambridge, UK; #ab113984; 1:1000) and ZEB1 (R&D Systems, Wiesbaden, Germany; #639914; 10 µg/ml). The next day, the PLA probe solution was added to the cells as described in the protocol. After the ligation and amplification steps, the nuclei were stained using ProLong® Gold Antifade reagent (Life Technologies, Darmstadt, Germany). Detection was performed using a Nikon Eclipse Ti-S fluorescence microscope (Nikon, Tokyo, Japan).

### Chromatin-immunoprecipitation (ChIP)

A detailed protocol with lists of used antibodies, respective dilutions, and primers was given in the Supplements. Briefly, ChIP was performed using the ChIP-IT High Sensitivity^®^ kit (Active Motif, La Hulpe, Belgium). Samples were analyzed by qPCR as previously described. A summary of analyzed gene regions is depicted in the Supplementary methods. Fold enrichment against the IgG control was calculated as previously described^15^.

### Immunofluorescence

Details were given in the Supplements. In brief, HCT cells were used for staining of F-Actin filaments and cells were mounted on object slides with ProLong^®^ Gold Antifade reagent (Life Technologies, Darmstadt, Germany). Confocal images were acquired using Carl Zeiss instruments (Carl Zeiss AG, Oberkochen, Germany). Images were edited using ZEN imaging software, Adobe PhotoShop CS5 and ImageJ software.

### Chorioallantoic membrane (CAM) assay

This method was performed as previously described^16^ and a detailed protocol is given in the Supplementary information. In short, fertilized eggs were opened at day 8 of embryonic development and resealed with sterile tape. On day 9, HCT cell lines (embedded in Matrigel) were placed onto the CAM. *In ovo* xenografts were further incubated for 5 days and tumor size was documented. Cells were used for IHC (FFPE) and protein/RNA analyses (fresh frozen).

### Bisulfite conversion and pyrosequencing analysis

A detailed protocol of the bisulfite conversion, the pyrosequencing methods, and the primer sequences can be found in the Supplement.

### Structural modeling of protein–protein interactions

Elaborate descriptions of protein modeling were placed in the Supplements. The following mathematical tools were used: MODELLER^17^, ITASSER^18^, ClusPro^19,20^, PIC^21^, and CHIMERA^22^.

### Bioinformatic workflow

A comprehensive explanation of the workflow was given in the Supplements. In brief, the distance between HCT116 cell lines was calculated using the Pearson correlation dissimilarity measure. Genome wide search for possible ZEB1 targets was performed using the TRANSFAC^®^ database (BIOBASE GmbH, Wolfenbüttel, Germany; Version 2015.3). Genes potentially regulated by p21 and ZEB1 were identified by combining the statistical PCR Array Dataset analysis and the TRANSFAC^®^ data.

### Statistical analyses

A detailed summary of the statistical analyses and the used patient data sets can be found in the Supplements.

## Results

### ZEB1 initiates EMT in p21 knockout cells

Epigenetic alterations such as DNA methylation and posttranslational modifications of histone proteins are crucial for the EMT program^23^. To identify how the EMT transcription factor ZEB1 cooperates with chromatin to trigger the EMT program we first defined an appropriate in vitro model. Recently, it has been shown by Li et al. that loss of the cell cycle inhibitor p21 leads to a more mesenchymal phenotype in HCT116 (HCT) cells. So first, we confirmed the spindle-shaped cell type by immunofluorescence staining for F-Actin (Supplementary Fig. S1A).

We could also verify a significant upregulation of Vimentin and ZEB1 as well as a significant decrease of E-Cadherin in a fraction of p21 knockout (p21–/–) cells (Supplementary Fig. S1B, C). Thus, we suggest that p21–/– cells have undergone a partial EMT showing a phenotypic heterogeneity and cellular plasticity with mesenchymal and epithelial characteristics. For this reason we further used the HCT116 p21–/– cells as an EMT model to investigate epigenetic ZEB1 signaling.

**Fig. 1:**
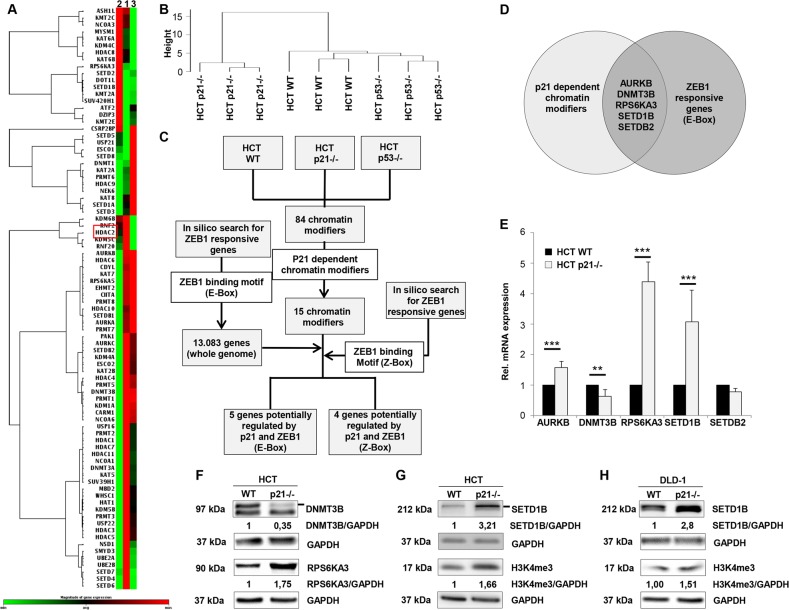
p21–/– cells show a dysregulation of chromatin modification enzymes. **a** Heat map representation of gene expressions (data sets of human chromatin modification enzymes) of HCT cell lines analyzed by RT² PCR Profiler Array; *n* = 3. **b** Hierarchical dendrogram clustering of samples from RT^2^ Profiler PCR Array data. **c** Combination of statistical analysis of RT² Profiler PCR Array Dataset and genome wide search for ZEB1 transcription factor binding sites (TFBS) using TRANSFAC^®^ software. **d**, Venn diagram of genes, that are significantly regulated by p21 and also possess a potential E-box TFBS for ZEB1 in their promoter regions. **e** RT-qPCR validation of potential target genes of ZEB1 and p21 for HCT WT and HCT p21–/– cells; *n* ≥ 3 (**p < 0.01; ***p < 0.001). **f** Representative western blot analysis of HCT WT cells (HCT WT, HCT p21–/–) cells for DNMT3B and RPS6KA3; *n* = 3. Fold expression is represented relative to GAPDH loading control; *n* = 3. **g** Representative western blot analysis of HCT WT cells (HCT WT, HCT p21–/–) cells for SETD1B and H3K4me3; *n* ≥ 2. Fold expression is represented relative to GAPDH loading control; *n* ≥ 2. **h** Representative western blot analysis of DLD-1 (DLD-1 WT, DLD-1 p21–/–) cells for SETD1B and H3K4me3; *n* ≥ 2. Fold expression is represented relative to GAPDH loading control; *n* ≥ 2. **e** mean ± s.d. Unpaired two-tailed Student’s *t*-test.

To identify chromatin modifying enzymes that are regulated in p21–/– cells we used a cDNA array with 84 different chromatin modifiers (Qiagen, PAHS-085Z). Since p21 is the major transcriptional target of the tumor suppressor p53, we also included HCT p53–/– cells to eliminate any p53 driven effects. The heatmap clustergramm (Fig. 1a) and hierarchical clustering (Fig. 1b) using the Pearson correlation dissimilarity as a measure to calculate the distance between the three different cell lines showed that the HCT WT and HCT p53–/– cells highly overlap in their gene expression pattern. Interestingly, gene expression pattern of HCT p21–/– cells differed remarkably from that of HCT WT and HCT p53–/– cells. In contrast to others^24,25^, HCT p53–/– cells show a cobble-stone like morphology and the expression levels of Vimentin or ZEB1 were comparable to the levels in HCT WT cells (Supplementary Fig. S1A, B). There was only a single gene (HDAC2) that significantly differed in gene expression (fold change ≥ ±2) between HCT WT and HCT p53–/– cells and was found to be 2-fold downregulated (*p* = 0.052).

Further analysis using the hclust function for differential expression signature and gene clusters (*p* < 0.05) was performed to evaluate unique genes in p21–/– cells and we identified 15 chromatin modification enzymes that fit our criteria (Table 1). Six of these genes (DOT1L, DZIP3, KMT2A, RPS6KA3, SETD1B, and SETD2) were upregulated and nine genes (AURKB, DNMT3B, ESCO2, HAT1, HDAC1, HDAC5, HDAC11, RPS6KA5, and SETDB2) were downregulated in HCT p21–/– cells compared to HCT WT cells.

**Table 1:** Dysregulated genes in HCT116 p21–/– cells compared to HCT116 WT cells.

Gene	Unigene no.	RefSeq no.	Fold Change	*p*-value	Function	Active/repressive code
*A: upregulated genes*						
DOT1L^a^	Hs.713641	NM_032482	1.6625	0.02893	Histone methyltransferase	Repressive
DZIP3^b^	Hs.409210	NM_014648	1.7171	0.02113	Histone ubiquitination	Repressive
KMT2A^c^	Hs.258855	NM_005933	1.5583	0.01501	Histone methyltransferase	Active
RPS6KA3^d^	Hs.445387	NM_004586	1.7695	0.01638	Histone phosphorylation	Active
SETD1B^e^	Hs.507122	NM_015048	1.7331	0.00850	Histone methyltransferase	Active
SETD2^f^	Hs.517941	NM_014159	1.4879	0.01639	Histone methyltransferase	Active
*B: downregulated genes*						
AURKB^g^	Hs.442658	NM_004217	−2.25532	0.00003	Histone phosphorylation	Active
DNMT3B^h^	Hs.713611	NM_006892	−2.17850	0.00497	DNA Methyltransferase	Repressive
ESCO2^i^	Hs.99480	NM_001017420	−2.77663	0.00497	Histone acetyltransferase	Active
HAT1^j^	Hs.632532	NM_003642	−2.05623	0.00621	Histone acetyltransferase	Active
HDAC1^k^	Hs.88556	NM_004964	−2.05148	0.03049	Histone deacetylase	Repressive
HDAC5^l^	Hs.3352	NM_001527	−3.76678	0.00621	Histone deacetylase	Repressive
HDAC11^m^	Hs.744132	NM_024827	−2.00926	0.00377	Histone deacetylase	Repressive
RPS6KA5^n^	Hs.510225	NM_004755	−2.06098	0.00621	Histone phosphorylation	Active
SETDB2^o^	Hs.631789	NM_031915	−2.40050	0.00621	Histone methyltransferase	Repressive

^a^DOT1-like, histone H3 methyltransferase (S. cerevisiae).

^b^DAZ interacting protein 3, zinc finger.

^c^Myeloid/lymphoid or mixed-lineage leukemia (trithorax homolog, Drosophila).

^d^Ribosomal protein S6 kinase, 90 kDa, polypeptide 3.

^e^SET domain containing 1B.

^f^SET domain containing 2.

^g^Aurora kinase B.

^h^DNA (cytosine-5-)-methyltransferase 3 beta.

^i^Establishment of cohesion 1 homolog 1 (*S. cerevisiae*).

^j^Histone acetyltransferase 1.

^k^Histone deacetylase 1.

^l^Histone deacetylase 5.

^m^Histone deacetylase 11.

^n^Ribosomal protein S6 kinase, 90 kDa, polypeptide 5.

^o^SET domain, bifurcated 2.

### ZEB1 affects the expression of chromatin modification enzymes

Next, we aimed to examine to which extent ZEB1 is responsible for the dysregulation of these chromatin modifiers in HCT p21–/– cells. We performed a genome wide search for human genes, which possess transcription factor binding sites of ZEB1, known as the E-box (5′-CANNTG-3′). After extracting 13,083 genes that gather a possible binding motif for ZEB1 by in silico analysis, we compared them with our selected gene pool of chromatin modifiers. We identified five genes that are potentially regulated by both, p21 and ZEB1 (Fig. 1c, d). Furthermore, we analyzed the ZEB1 Z-Box transcription factor binding sites (5′-CAGGTG-3′ or 5′-CAGGTA-3′) in 15 p21 chromatin modifiers and it was observed that four genes out of 15 p21 chromatin modifiers contained the motif. The genes are: DNA (cytosine-5-)-methyltransferase 3 beta (DNMT3B), SET domain containing 1B (SETD1B), Histone Deacetylase 5 (HDAC5), and Histone Deacetylase 11 (HDAC11). In RT-qPCR and western blotting we confirmed the upregulation of two chromatin modifiers (SETD1B—Histone-Lysine N-Methyltransferase, RPS6KA3—Ribosomal Protein S6 Kinase A3) in the p21–/– cell line and the downregulation of the de novo methyltransferase DNMT3B (Fig. 1e–g).

The expression level of AURKB was found to be up-regulated in RT-qPCR, whereas it was downregulated in the array analysis. Due to this contradictory finding we excluded AURKB from our study. We further focused on SETD1B since its role in EMT regulation is nearly unknown. SETD1B is responsible for the trimethylation of lysine 4 of the histone H3 subunit (H3K4me3), which is a specific tag for genetic transcriptional activation. There was an increase in SETD1B and corresponding H3K4me3 protein levels in western blot analysis not only in HCT p21–/– cells (Fig. 1g), but also in colorectal DLD-1 p21–/– cells (Fig. 1h).

**Fig. 2:**
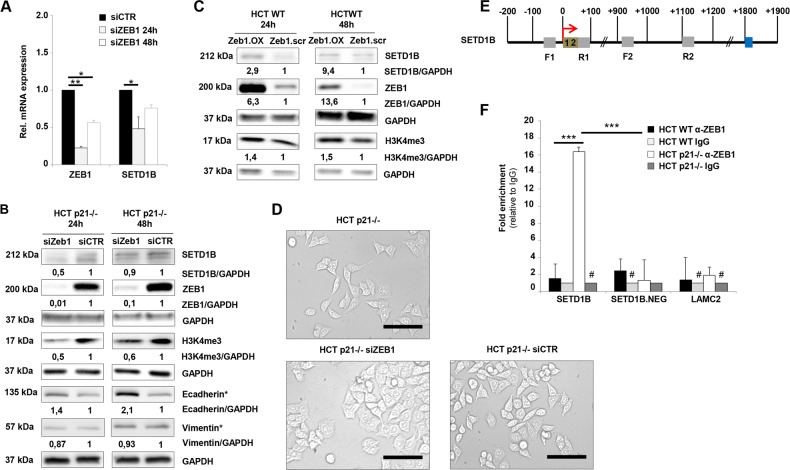
ZEB1 affects the expression of chromatin modification enzymes. **a**, **b** RT-qPCR analysis of SETD1B and representative western blot analysis of E-Cadherin, Vimentin, SETD1B, and H3K4me3 in HCT p21–/– cells upon ZEB1 knockdown after siRNA transfection; *n* = 3 (*p < 0.05; **p < 0.01). Fold expression (western blot) is represented relative to GAPDH loading control; *n* = 3. *Samples were loaded on the same membrane **c** Representative western blot analysis of SETD1B and H3K4me3 in HCT WT cells upon ZEB1 overexpression after transfection; *n* = 3. Fold expression is represented relative to GAPDH loading control; *n* = 3. **d** Representative phase contrast images of HCT p21–/– cells and of HCT p21–/– cells upon ZEB1 knockdown after siRNA transfection for 24 h; Phase contrast magnification = 200×; Scale bar = 100 µm; *n* ≥ 4. **e** Schematic display of promoter regions for SETD1B with respective ZEB1 E-Box binding sites (olive green), ZEB1 Z-Box binding site (blue), primer pairs (gray) and transcription start site (TSS) (red arrow). **f** ChIP analysis of ZEB1 binding capacity to SETD1B promoter region showing an increase of ZEB1 binding after loss of p21; *n* = 3 (***p < 0.001). **a**, **f** Mean ± s.e.m. Unpaired two-tailed Student’s *t*-test.

To examine if ZEB1 is responsible for transcriptional regulation of SETD1B, we performed a transient ZEB1 knockdown in HCT p21–/– cells and studied the expression of SETD1B using RT-qPCR and Western blot analysis (Fig. 2a, b). Indeed, we observed a significant downregulation of ZEB1 at 24 h after siRNA transfection on the mRNA level and at 48 h on the protein level. Moreover, H3K4me3 protein level was also downregulated after siRNA transfection (Fig. 2a, b). In accordance with this finding, Western blot analysis showed that ZEB1 overexpression led to a significant increase in expression of SETD1B and its associated code H3K4me3 at 24 h and also at 48 h compared to the vector control (Fig. 2c). Under ZEB1 si transfection we observed a remarkable increase in E-Cadherin protein levels accompanied by a loss in spindle cell morphology with a shift into more cobble stone like morphology although the Vimentin protein levels did not change (Fig. 2b, d).

Finally, chromatin immunoprecipitation (ChIP) analysis demonstrated an enrichment of ZEB1 at the E-Box containing promoter region of SETD1B (Fig. 2e, f). This binding was significantly reinforced in p21–/– EMT cells but not at a distant region downstream of the transcription site that does not contain an E-box element (Fig. 2e, f). For the first time, we show in vitro that the chromatin modifier SETD1B is a direct transcriptional target of ZEB1. This is very remarkable finding as ZEB1 has been shown to mainly act as a transcriptional repressor of epithelial genes^4^. Hence, our findings provide another example for ZEB1 as an inducer of gene transcription^26,27^.

### p21–/– cells shown an upregulation of the ZEB1 and SETD1B in vivo

**Fig. 3:**
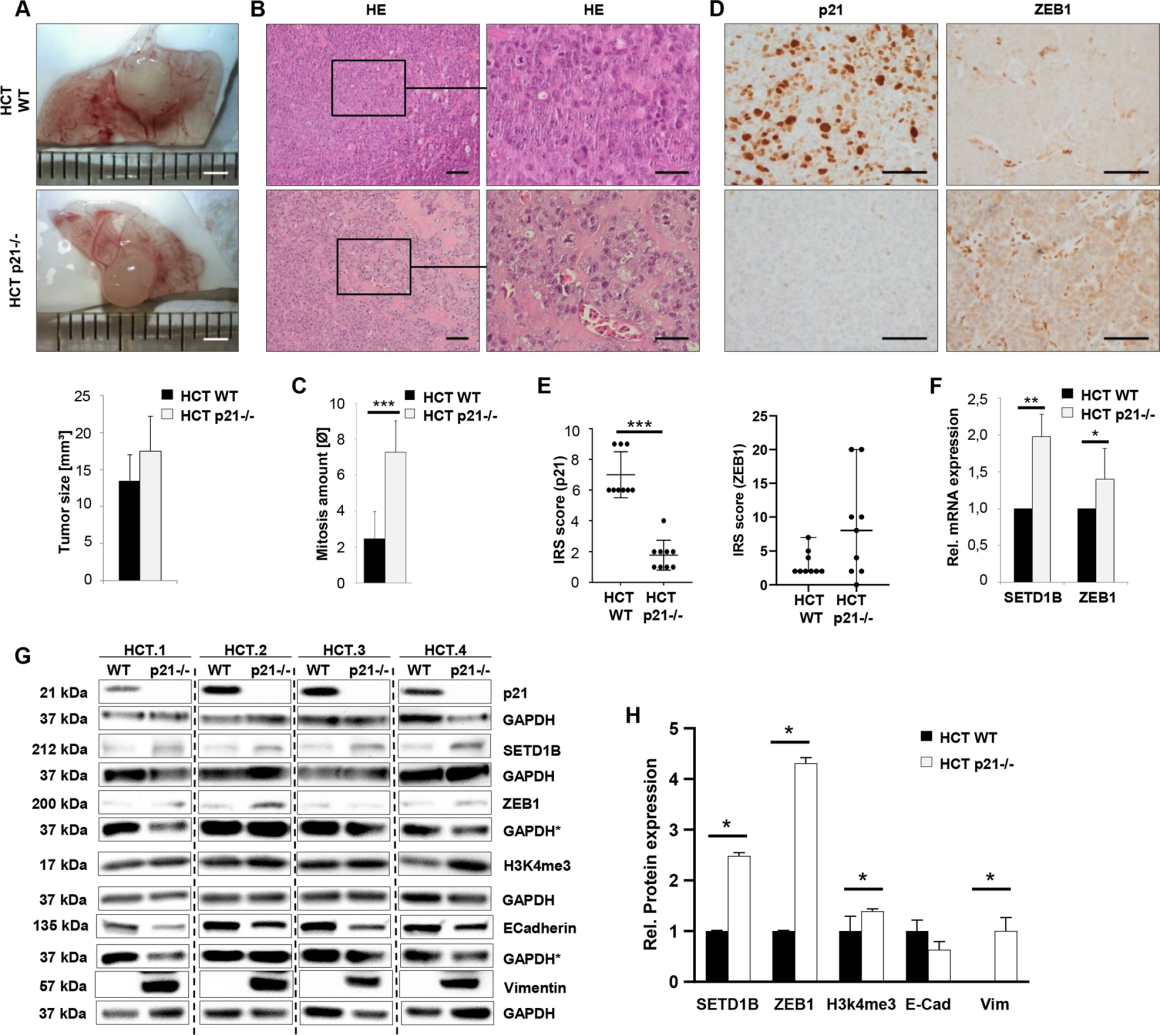
p21–/– cells show an upregulation of ZEB1 and SETD1B in vivo. **a** Representative light microscopy images of fixed and extracted CAM tumors (HCT WT and HCT p21–/–); Magnification = 100×; Scale bar = 2 mm; *n* ≥ 8. Tumor volume calculation in HCT CAM tumors (HCT WT and HCT p21–/–); *n* ≥ 8. **b** Representative immunohistochemical stainings on FFPE CAM sections for HE (Magnification = 200× and 600×); Scale bar = 100 and 50 µm, *n* ≥ 8 and **c** Tumor mitosis calculation in HCT CAM tumors (HCT WT and HCT p21–/–); *n* ≥ 8 (***p < 0.001). **d** Representative immunohistochemical stainings on FFPE sections for p21 and ZEB1 of CAM tumors (HCT WT and HCT p21–/–); Magnification = 400×; Scale bar = 100 µm; *n* ≥ 8. **e** Dot plot analysis of expression intensities (IRS score) for immunohistochemical stainings of p21 and ZEB1 of CAM tumors (HCT WT and HCT p21–/–), *n* ≥ 8 (***p < 0.001). **f** RT-qPCR analysis of unfixed CAM tumors tissue for SETD1B and ZEB1; *n* = 4. (*p < 0.05; **p < 0.01). **g** Western blot analysis of unfixed CAM tumor samples confirming in vitro data; *n* = 4. *The same membrane was used for both proteins. **h** Protein expression measured by western blot of unfixed CAM tumor samples; *n* = 4 (*p < 0.05). Fold expression is represented relative to GAPDH loading control; *n* = 4. **a**, **c**, **e**, **h** mean ± s.d. Unpaired two-tailed Student’s *t*-test. **f** mean ± s.e.m. Unpaired two-tailed Student’s *t*-test.

Since 2D culture fails to reflect the architectural features of native tissues mainly without considering the effects of extracellular matrix, we investigated, if ZEB1 is also able to regulate SETD1B and its corresponding active histone mark in vivo. We used the chorioallantoic membrane (CAM) assay (Fig. 3a). When evaluating CAM xenografts we observed that HCT p21–/– cells formed more aggressive tumors with larger size, an infiltrative growing pattern at the invasion front, and higher proliferation rate (Fig. 3a–c). We then performed immunostainings for p21 and ZEB1, in formalin-fixed and paraffin embedded CAM xenografts of HCT WT and HCT p21–/– cells (Fig. 3d). Xenografts of p21–/– cells showed higher ZEB1 staining scores compared to HCT WT cells when scoring the staining intensity (Fig. 3e).

In HCT WT cells ZEB1 was slightly expressed only in single cells, thus a score = Null has never been reached. Since no suitable antibody for immunostaining of SETD1B was available we analyzed SETD1B by RT-qPCR and western blot analysis of freshly prepared CAM xenograft samples (Fig. 3f). Its corresponding H3K4me3 code was also investigated by western blotting (Fig. 3g, h). We verified the high expression of ZEB1 and SETD1B in p21–/– xenografts as observed in vitro. As expected there was a significant upregulation of the H3K4me3 code in EMT cells (Fig. 3g, h).

In a next step, we analyzed the set of upregulated and downregulated chromatin modifying enzymes (CMEs) summarized in Table 1 using the STRING database to better understand the connections between the individual CMEs. We included ZEB1 into the analysis in order to highlight already established or predicted interactions between ZEB1 and our targets of interest (Supplementary Fig. S1D, E). Although some links between several CMEs exist, so far ZEB1 mediated interactions with chromatin modifiers have not been validated.

**Fig. 4:**
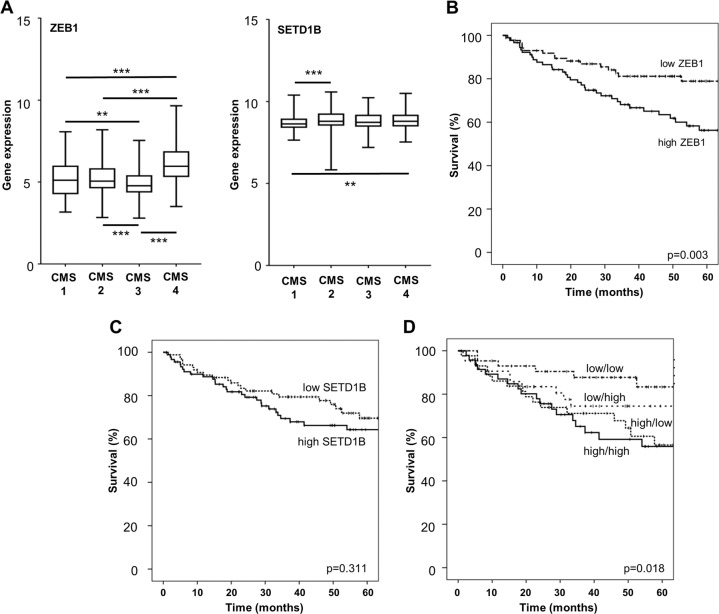
Relevance of ZEB1-SETD1B network in human colorectal cancer. **a** Box plot representation of SETD1B and ZEB1 expression in the four CMS colorectal cancer subtypes (**p ≤ 0.01, ***p ≤ 0.001). *n* = 944 (GSE13067, GSE13294, GSE14333, GSE17536, GSE20916, GSE2109, GSE33113, GSE35896 and GSE42284). **b**–**d** Kaplan–Meier-curve to correlate gene expression and patient´s survival data, *p*-value by log rank test; ZEB1 (**b**), SETD1B (**c**) combination score of ZEB1/SETD1B (**d** low/high—low ZEB1/high SETD1B, high/low—high ZEB1/low SETD1B); stratification into groups by median values of gene expression, *n* = 177 (GSE17536).

Furthermore, we increased the clinical data set using the 944 colon cancer patients from the consensus molecular subtype study^28^. First, we found a high and significant correlation between ZEB1 and SETD1B (*r*_s_ = 0.523, *p* < 0.001) in this tumor group. Second, we confirmed that SETD1B and ZEB1 showed indeed highest expression levels in the mesenchymal CMS4 subtype (Fig. 4a).

To further examine the prognostic value of ZEB1/SETD1B axis in colon cancer patients, we investigated a data set of 177 patients with colorectal carcinoma (GEO DataSet: GSE17536) (Fig. 4b–d). First, we defined a high and low expressing group using the median value for each parameter. Patients who had tumors with high ZEB1 expression had a worse prognosis than those having tumors with low ZEB1 expression (logrank *p* = 0.003, Fig. 4b). After 5 years, patients affected by tumors with high ZEB1 and those with low ZEB1 had overall survivals of 56 and 78.9%, respectively. SETD1B did not show clinical relevance in Kaplan–Meier analysis (Fig. 4c). When combining both parameters, we recognized that high SETD1B/high ZEB1 expression seems to be useful for defining a high risk subgroup of tumors (logrank *p* = 0.018; overall survivals = 47.5%; Fig. 4d) with unfavorable prognosis whereby ZEB1 seems to be the more dominant factor. Low SETD1B/high ZEB1 cases (*n* = 43) showed an overall survival of 56.5% and high SETD1B/low ZEB1 (*n* = 43) presented an overall survival of 74.5%. Interestingly 83.3% of the 43 patients from the low/low group survived after 5 years of follow-up. Thus, we suggest that the ZEB1-SETD1B axis has strong prognostic value for CRC patients.

### ZEB1 induces a SETD1B dependent positive feedback loop

**Fig. 5:**
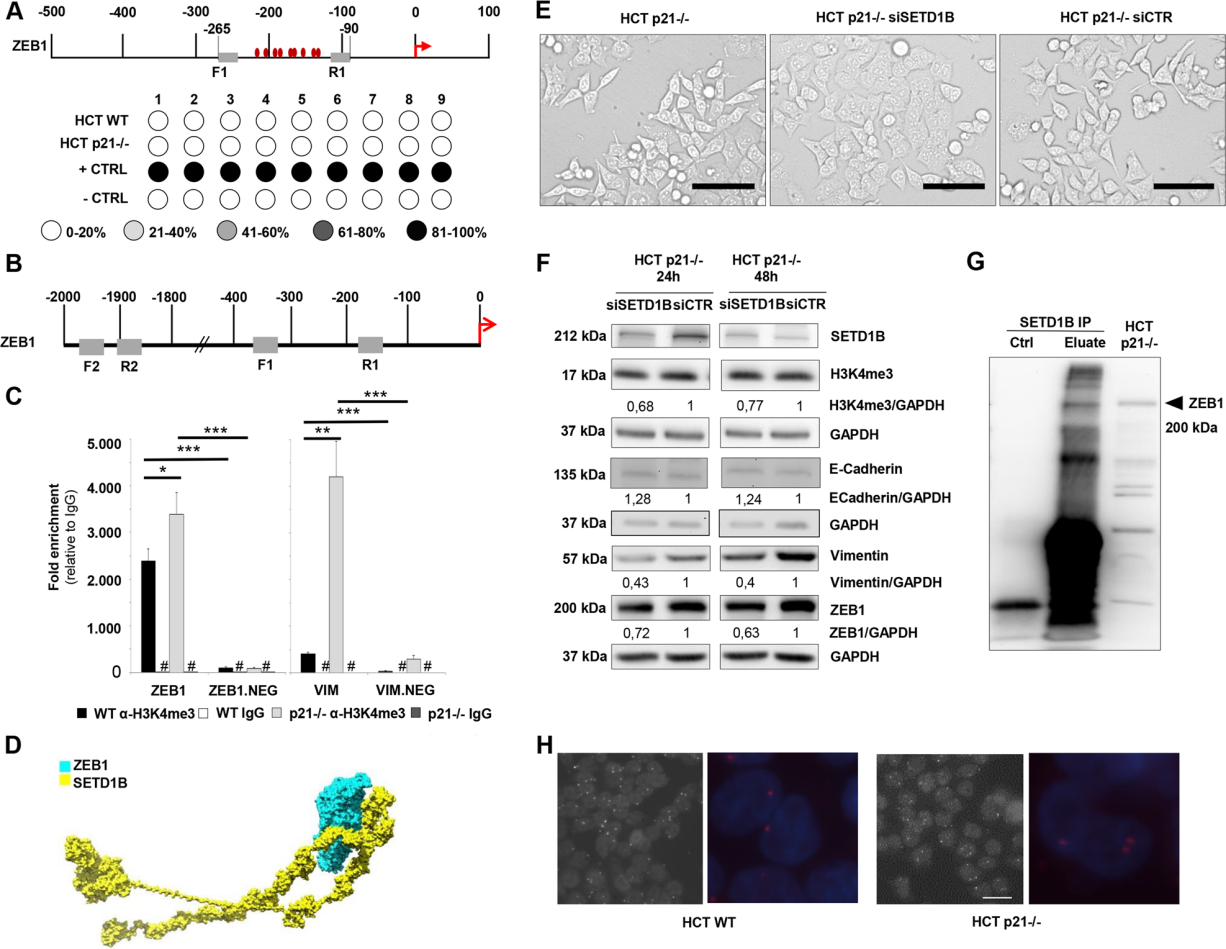
SETD1B reinforces ZEB1 expression. **a** Representative pyrosequencing analysis of HCT cell lines for the CpG island in the ZEB1 promoter region; *n* ≥ 2. This data shows that the ZEB1 promoter is hypo-methylated in both cell lines, indicating that the loss of p21 does not affect the methylation status of the ZEB1 promoter and hence the transcriptional upregulation of ZEB1 is not caused by demethylation. **b** Schematic display of promoter regions for ZEB1 with respective primer pairs (gray) and transcription start site (TSS) (red arrow). **c** ChIP analysis of H3K4me3 binding capacity to Vimentin and ZEB1 promoter region; *n* = 3 (*p < 0.05). **d** The complex of (ZEB1-SETD1B) was formed by docking the individual proteins using the Protein–Protein docking tool ClusPro^19,20^. The complex with the least energy was chosen for the analysis of interactions between the proteins considered. The cluster scores of the complexes from the ClusPro server were used to understand the energy profiles of the (ZEB1-SETD1B) complex. The ionic, hydrophobic, hydrogen bond interactions were identified and analyzed using the protein interaction calculator (PIC)^21^ and are summarized in the Supplements. All renderings were done using CHIMERA^22^. **a** CpGenome universal methylated DNA (MerckMillipore) was used as a positive control and unmethylated human placenta DNA was used as a negative control. **c** mean ± s.e.m. Unpaired two-tailed Student’s *t*-test. **e** Representative phase contrast images of HCT p21–/– cells and of HCT p21–/– cells upon SETD1B knockdown after siRNA transfection for 24 h; Phase contrast magnification = 200×; Scale bar = 100 µm; *n* ≥ 4. **f** Representative western blot analysis of SETD1B, E-Cadherin, Vimentin, ZEB1, and H3K4me3 in HCT p21–/– cells upon SETD1B knockdown after siRNA transfection. Fold expression (western blot) is represented relative to GAPDH loading control; *n* ≥ 2. **g** Co-IP of endogenous SETD1B and ZEB1 in HCT p21–/– cells showing co-precipitated ZEB1 after SETD1B immunoprecipitation. HCT p21–/– cell lysates were used as a control lysate; Ctrl IgG control. **h** Representative images of proximity ligation assay for SETD1B and ZEB1 in HCT WT and HCT p21–/–; scale: 25 µm; 400× magnification, fluorescence image (blue: DAPI to visualize the nucleus, red: to visualize the protein complex) is computer enlarged. White/red dots represent the protein interaction.

Recently, it has been shown that an interaction between the EMT transcription factor TWIST1 and the chromatin modifier NSD2, a histone methyltransferase, is crucial for transcriptional regulation of TWIST1 itself in prostate cancer^29^. Thus, we wanted to analyze if promoter demethylation or SETD1B-dependent histone mark could be responsible for the transcriptional upregulation of ZEB1 itself in EMT cells. For this, we examined 7 CpG islands in the ZEB1 promoter 100–300 bp upstream of the transcription site (Fig. 5a). Both cell lines were unmethylated for this region suggesting that ZEB1 transcriptional upregulation is not caused by demethylation of its promoter. Next, we again chose ChIP experiments to examine the chromatin state at the ZEB1 promoter. Interestingly we were able to show an increase in H3K4me3 methylation in the promoter region of ZEB1 in p21–/– cells compared to the WT cells (Fig. 5b, c), Presence of this active histone mark is consistent with increased ZEB1 expression in p21–/– cells. Next we performed a SETD1B si transfection in HCT116 p21–/– cells. We observed an effective downregulation of SETD1B protein and the corresponding H3K4me3 code at 24 h. ZEB1 protein levels were decreased at 24 and 48 h, whereas the E-Cadherin protein levels were nearly unchanged. Interestingly the Vimentin protein levels decreased at both time points (Fig. 5f). To analyze if the SETD1B mediated active histone code is associated with the high Vimentin expression in p21–/– cells we extended the ChIP experiment. Indeed, we confirmed an enrichment of the H3K4me3 code at the Vimentin promoter with a concomitant promoter demethylation (Fig. 5c and Suppl. Fig. 2).

Since it is known that transcriptional activity of ZEB1 is mainly mediated by its recruitment of co-repressors or co-activators we were led to the question if ZEB1 might recruit SETD1B into a protein complex. Towards this, we modeled the complex of (ZEB1-SETD1B) and analyzed the energy profiles and interactions between these two proteins (Fig. 5d) in HCT and HCT p21–/– cells. Our structural modeling showed that the binding energy of the (ZEB1-SETD1B) complex was decreased by ~20% in p21–/– cells (−1410.5 kcal/mol) compared to the energy of the complex in HCT cells (−1137.5 kcal/mol) showing a stabilization of this complex in EMT cells. Several hydrophobic amino acid residues (730–786) in the Proline rich regions of SETD1B were found to interact with Threonines, Leucines, Cysteines, and Methionines in the region between the Zinc finger domain and homeobox domain of ZEB1 and between the homeobox domain and the second zinc finger domain of ZEB1 (data not shown). We performed a co-immunoprecipitation experiment using the SETD1B antibody and detected ZEB1 in the precipitate (Fig. 5g). Vice versa using two different ZEB1 antibodies we were not able to show any SETD1B signal in the precipitate. Nevertheless, proximity ligation assay verified the existence of the protein complex between SETD1B and ZEB1 in HCT and HCT p21–/– cells as proposed in the in silico modeling (Fig. 5h). As a negative control we used a membrane bound protein, EpCAM, and did not find any signals of SETD1B and EpCAM binding in both cell lines showing the specificity of the experimental procedure (Fig. 5h). Since we did not detect significantly more interaction signals in p21–/– cells we suggest that the additional ZEB1 and SETD1B molecules might also act in other protein complexes independent of the already existing ZEB1/SETD1B protein complex. In summary, our findings support the assumption that ZEB1 selectively recruits chromatin modifiers in a promoter-specific manner.

**Fig. 6:**
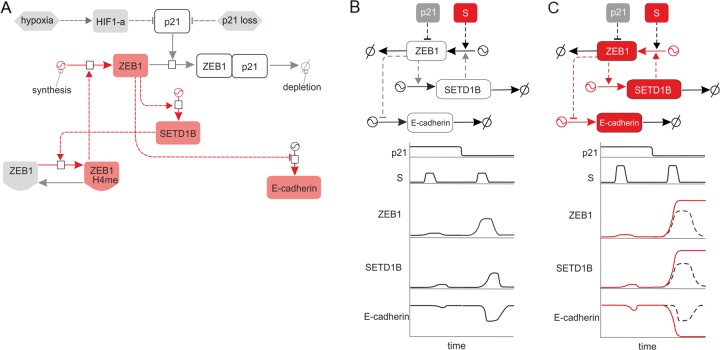
ZEB1 regulates SETD1B via a regulatory circuit integrated by feedback and feedforward loops. **a** This system recapitulates in a regulatory network all the interactions relevant for the interplay between SETD1B and ZEB1 in the context discussed. The key element of the system is the double positive feedback loop between ZEB1 and SETD1B (ZEB1 → SETD1B → ZEB1/H3K4me3 → ZEB1). Through this loop each protein can reinforce the activity/expression of the other. This can amplify the activation of the circuit. **b** Under low transient stimulation (S) of ZEB1, the circuit responds with transient, low level activation even in the absence of p21. **c** The structure of the circuit suggests a medium but transient stimulation (S) of ZEB1. This activation can provoke an irreversible enhancement in the expression and activity of both molecules via triggering of SETD1B and feedback-regulating ZEB1 activation. This in turn may trigger the EMT phenotype by consistent, long-lasting inhibition of epithelial marker E-Cadherin. In solid red lines, we represent the long-lasting activation of the circuit, while the black dashed lines represent the transient activation of the circuit that would appear in case of disruption of the positive feedback loop.

Figure 6a recapitulates in a regulatory network all the interactions relevant for the regulation of SETD1B in the context discussed. The key element of the system is the double positive feedback loop between ZEB1 and SETD1B (ZEB1 → SETD1B → ZEB1/H3K4me3 → ZEB1). Through this loop each protein can reinforce the activity/expression of the other. This can amplify the activation of the circuit: small/transient activation of ZEB1 can provoke strong/permanent activation of the circuit. Additionally, the network includes a coherent feedforward loop. In this loop, ZEB1 directly promotes the expression of SETD1B expression. The different regulation patterns of the ZEB1-SETD1B circuit under the wildtype (Fig. 6b) and tumorigenic (Fig. 6c) conditions are shown as discussed. In the wildtype case, the system works as a coherent feedforward regulating the expression of SETD1B and can act as a noise buffer system under ZEB1 signaling noise. In solid lines, we represent how short-duration, low-intensity stimulation triggers a short-duration, low-intensity activation of ZEB1 and SETD1B. In the scheme on the right under tumorigenic conditions (Fig. 6c) that promote an overexpression or overactivation of ZEB1, the positive feedback loop between ZEB1 and SETD1B can get triggered. This would induce an irreversible enhancement in the expression and activity of both molecules under transient stimulation of ZEB1 (solid lines). Under these conditions, the coherent feedforward loop gets activated. This behavior is totally different to that of the system under physiological conditions, represented in dashed lines.

## Discussion

With this study, we describe that ZEB1 causes severe alterations in the expression patterns of chromatin modifying enzymes. We report that the ZEB1-mediated upregulation of histone methyltransferase SETD1B is stabilizing ZEB1-mediated EMT through different feedback mechanisms, in which the key element seems to be a positive feedback loop between ZEB1 and SETD1B. Through this loop each protein can reinforce the activity/expression of the other. Thus, our findings fit very well with the concept of EMT induction as a programmed epigenetic switch^30^.

The EMT transcription factor ZEB1 has multiple functions and if it is acting as a transcriptional repressor or activator strongly depends on the tumor type. There is a variety of co-repressors or co-activators that ZEB1 is interacting with. P300, DOT1L, Tip60, and PCAF are histone acetyltransferases that are recruited to activate gene expression, whereas interactions with CtBP, HDAC1, and SIRT1 are rather repressing gene expression^10,11,26,31^. Interestingly, treatment with HDAC inhibitors was found to effectively suppress EMT^32,33^.

Other ZEB1 interaction partners are the H3K4 demethylase LSD1 and BRG1 as a subunit of the SWI/SNF complex^33,34^. In accordance with and highly conclusive from these data we showed that ZEB1 induction in the mesenchymal HCT p21–/– cells led to a massive dysregulation of epigenetic players. Yet, there was no obvious preference for chromatin modifiers that are majorly activating (upregulated: KMT2A, RPS6KA3, SETD1B, and SETD2; downregulated: AURKB, ESCO2, HAT1, and RPS6KA5) or repressing factors (upregulated: DOT1L, DZIP3; downregulated: DNMT3B, HDAC1, HDAC5, HDAC11, and SETDB2). In our study we focused on SETD1B, a histone methyltransferase that contributes to the epigenetic control of chromatin structure by specifically methylating Lys4 at histone H3.

It is known that mutations of SETD1B play a role in tumorigenesis of gastric and colorectal cancers with MSI^35^. When we analyzed the mutation frequency of the 526 colorectal cancer patients reported in the TCGA PanCancer Atlas (www.cbioportal.org) we found 6% of SETD1B mutant cases (36 samples) with 26 cases had a mutation load higher than 1000. Recurrent missense mutations were reported in known protein domains such as the RNA recognition motif, the N-SET domain and the catalytic SET domain. Nevertheless, the pathogenic role of the single mutation variants and their clinical significance remain mostly unclear. It also remains unclear, which steps of cancer development are affected or regulated by SETD1B. Moreover, there is only speculation about SETD1B´s role in cancer and EMT. From clinical data we extracted the worst prognosis when both, ZEB1 and SETD1B were highly expressed whereas in the low/low group nearly all colorectal cancer patients survived the first 5 years after surgery. For endometrial carcinomas it was reported that for SETD1B mutant cases a higher myometrial invasion can be predicted^36^. In clear cell renal cell carcinomas SETD1B overexpression could discriminate metastatic from non-metastatic tumors^37^.

The SETD1B specific epigenetic modification of a trimethylated lysine 4 at histone 3 (H3K4me3) is well-accepted as a marker for transcriptionally active gene promoters. The observed enrichment of the H3K4me3 histone modification was induced by ZEB1-dependent induction of SETD1B expression at the ZEB1 promoter itself showing a novel feedback reinforcement loop for ZEB1. Furthermore, SETD1B seems to trigger the expression of Vimentin creating an active chromatin status with concomitant demethylation of the Vimentin gene promoter. Also, for bivalent genes during ES differentiation to neuronal precursor cells the H3K4me3 modification is known to become enriched at specific genes^38^. Our finding of a higher H3K4me3 code in EMT cells would fit with others who showed that an ablation of the H3K4 demethylase LSD1 in breast carcinoma cells led to an increased migration and metastasis^39^. In colorectal cancer, alterations in H3K4me3 levels were shown to be associated with tumor initiation^40^. The SETD1B-H3K4me3 epigenetic axis has been reported to contribute to increased iNOS expression in tumor-induced myeloid-derived suppressor cells to render T cell low responsive to antigen stimulation^40^. There is a very similar regulatory cascade in leukemia cells, where transcription factor NFkB recruits the MLL1 histone methyltransferase complex to activate NFkB target genes after TNF treatment^41^. It was previously reported that SETD1B is a possible causative gene for the pathogenesis of the 12q24.3 deletion syndrome^42,43^. In this regard patients with de novo SETD1B mutations showed signs of epilepsy, developmental delay, intellectual disability, and autism^44^. From these few available literature reports we suggest that the regulation of the H3K4me3 mark is extremely complex and an increase of SETD1B expression could affect many different pathways.

In this study, we verified that ZEB1 acts in concert with SETD1B to direct EMT an important cellular plasticity program. For the first time, we give evidence that ZEB1 directly binds to SETD1B in a protein complex. In the p21–/– cells this complex seems to be redirected to EMT-associated promoters. We strongly believe that mesenchymal p21–/– cells represent a suitable model for mechanistic EMT-related studies leading here to the identification of our novel molecular network. Since our analysis tool was an isogenic tumor cell line originating from a microsatellite instable tumor the clinical data^28^ strengthen our results also for MSS tumors that are summarized in molecular subtypes CMS2, CMS3, and CMS4.

## Conclusions

Here we describe a novel link between the EMT transcription factor ZEB1 and chromatin modification. For the first time we report that ZEB1 directly regulates the expression pattern of the histone methyltransferase SETD1B in colorectal cancer cells. Mechanistically we show that ZEB1 binds the promoter region of the SETD1B gene and that SETD1B dependent active H3K4me3 histone code seems to open the ZEB1 promoter, forming a positive feedback loop. Our study indicates a new example of an activator role of ZEB1 for one of the diverse functions that tumors acquire during EMT. Furthermore, we demonstrate pathophysiological relevance of our findings.

## Supplementary information

Suppl. Figure 1

Suppl. Figure 2

Suppl. Figure 3

Suppl. Figure Legends

Suppl. Material and Methods

Suppl. Tables

## Data Availability

The RT² Profiler PCR Arrays Data Set (PAHS-085Z) was submitted to the Gene Expression Omnibus (GEO) data repository as a Sub-Series under the accession number GSE107664 (PAHS-085Z). Data availability is given in Materials and Methods as well as Supplementary section.
