# Supplementary figures and images for: EMT transcription factor ZEB1 alters the epigenetic landscape of colorectal cancer cells
Source: Cell Death Dis. 2020 Feb 24;11(2):147. doi: 10.1038/s41419-020-2340-4 (PMC7040187; doi:10.1038/s41419-020-2340-4)

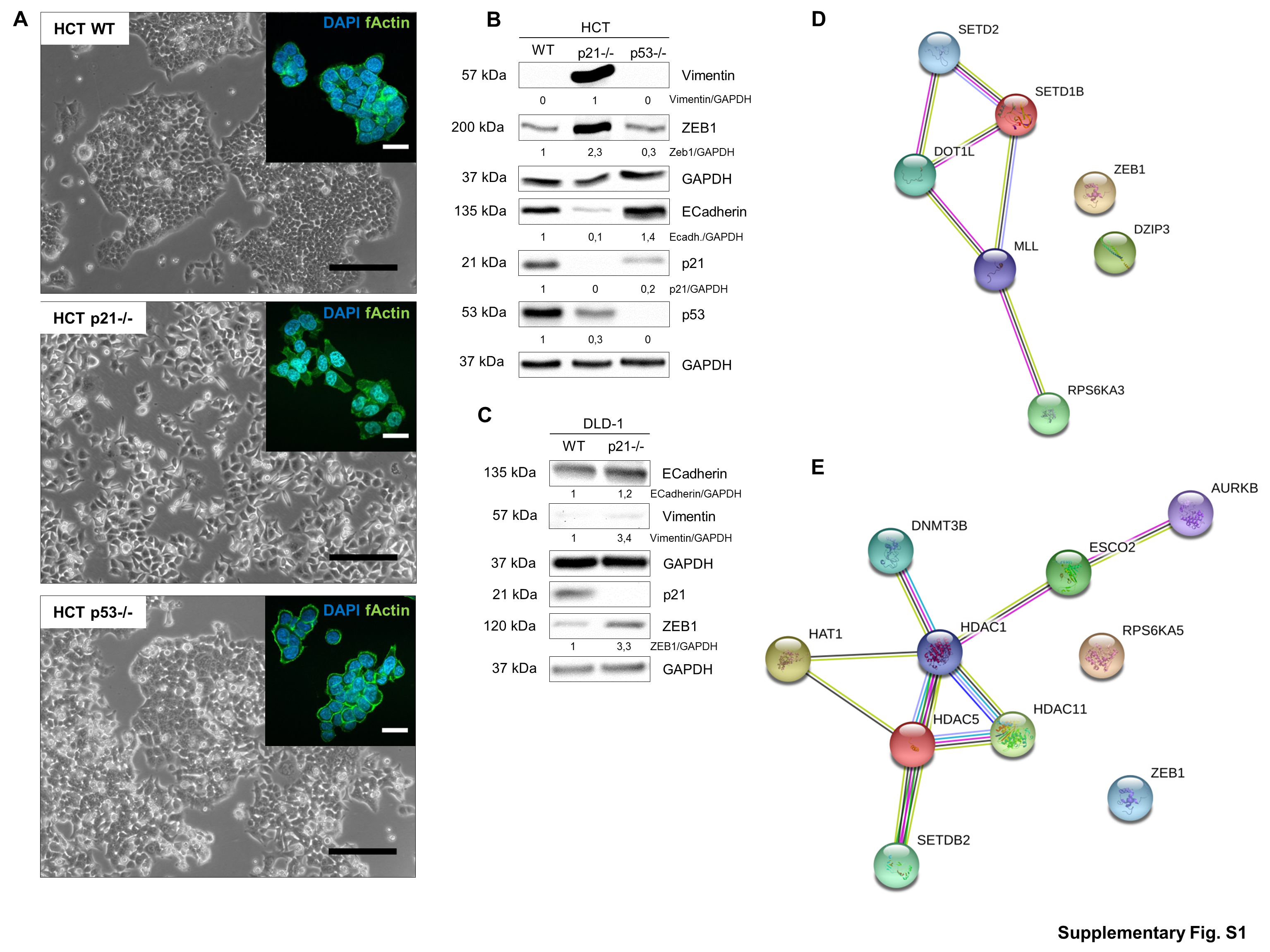

Supplement: Supplementary file 1 — Suppl. Figure 1 [file 41419_2020_2340_MOESM1_ESM.tif]

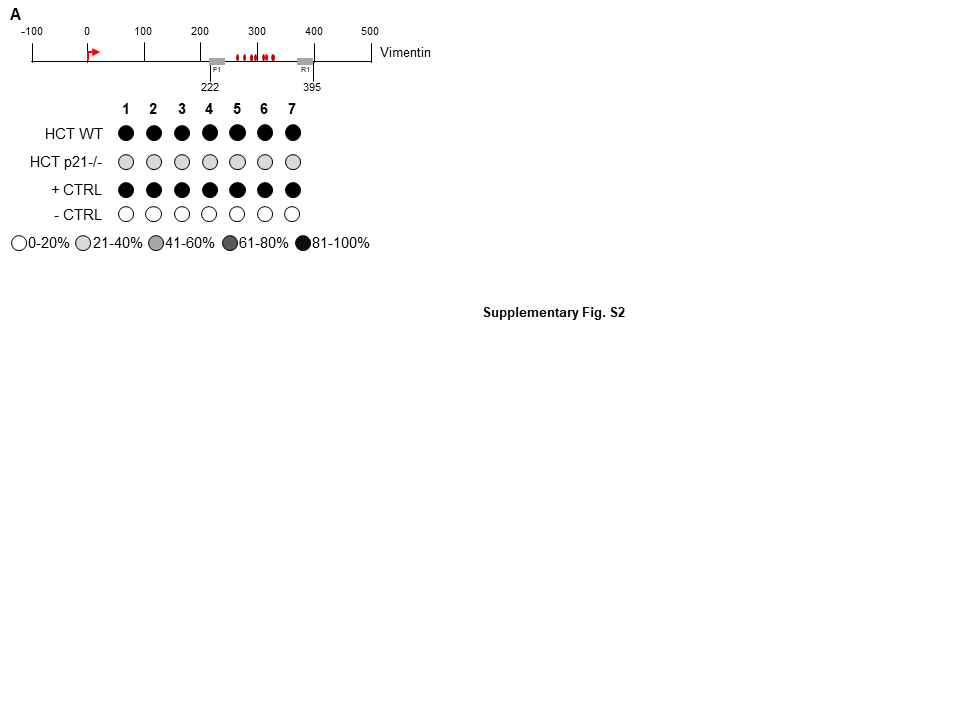

Supplement: Supplementary file 2 — Suppl. Figure 2 [file 41419_2020_2340_MOESM2_ESM.tif]

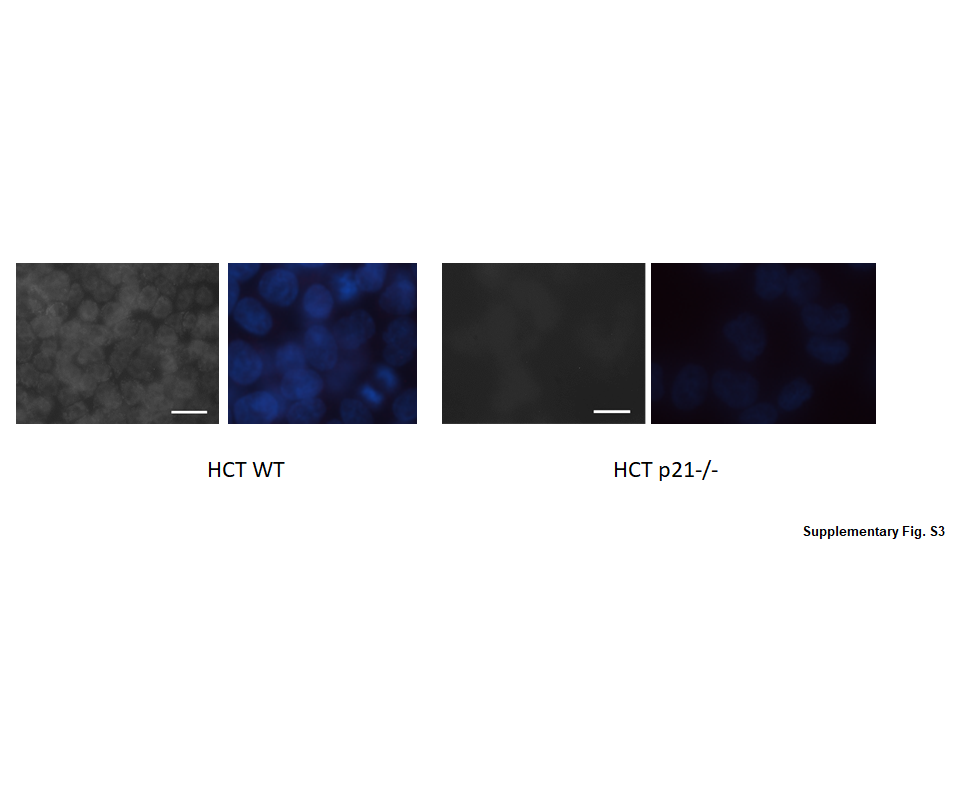

Supplement: Supplementary file 3 — Suppl. Figure 3 [file 41419_2020_2340_MOESM3_ESM.tif]
