# Supplementary material for: EMT transcription factor ZEB1 alters the epigenetic landscape of colorectal cancer cells
Source: Cell Death Dis. 2020 Feb 24;11(2):147. doi: 10.1038/s41419-020-2340-4 (PMC7040187; doi:10.1038/s41419-020-2340-4)
Supplement: Supplementary file 4 — Suppl. Figure Legends [file 41419_2020_2340_MOESM4_ESM.docx]

**Supplementary Legends**

**Supplementary Figure S1**

**ZEB1 initiates EMT in p21 -/- cells**

**A,** Representative phase contrast and immunofluorescence confocal microscopy images of HCT WT and HCT p21-/- cells; Phase contrast magnification = 100x, Confocal magnification = 630 x; Scale bar (Phase contrast) = 200 µm, Scale bar (Confocal) = 20 µm; *n* ≥ 4. **B,** Representative western blot of HCT WT, HCT p53-/-, and HCT p21-/- cells for genetic background and EMT markers; *n* ≥ 3. Fold expression is represented relative to GAPDH loading control; *n* ≥ 3. **C,** Representative western blot of DLD-1 (WT and p21-/-) for genetic background and EMT markers; *n* ≥ 2. Fold expression is represented relative to GAPDH loading control; *n* ≥ 2. **D-E,** STRING Data Base analysis for upregulated (D) and downregulated (E) CME genes in combination with ZEB1 showing the following connections: i) based on curated databases (turquoise); ii) determined through experiments (pink), iii) predicted interactions by gene neighborhood (dark green); by gene fusions (red); by gene co-occurrence (dark blue) and iv) by text mining (light green); by co-expression (black); by protein homology (purple).

**Supplementary Figure S2**

**The loss of p21 induces demethylation of the Vimentin promoter**

**A,** Representative pyrosequencing analysis of HCT cell lines for the CpG island in the Vimentin promoter region. This data shows that the Vimentin promoter is hypo-methylated in the p21-/- cell lines indicating that the loss of p21 does affect the methylation status of the Vimentin promoter and hence the transcriptional upregulation of Vimentin might be caused by demethylation.

**Supplementary Figure S3**

**No protein interaction between SETD1B and EpCAM**

Representative images of proximity ligation assay for SETD1B and EpCAM in HCT WT and HCT p21-/- cells; scale: 25µm; 400x magnification, fluorescence image (blue: DAPI to visualize the nucleus, red: to visualize the protein complex) is computer enlarged.
