# Supplementary material for: EMT transcription factor ZEB1 alters the epigenetic landscape of colorectal cancer cells
Source: Cell Death Dis. 2020 Feb 24;11(2):147. doi: 10.1038/s41419-020-2340-4 (PMC7040187; doi:10.1038/s41419-020-2340-4)
Supplement: Supplementary file 5 — Suppl. Material and Methods [file 41419_2020_2340_MOESM5_ESM.docx]

**Supplementary Information – Materials and Methods**

**Cell culture**

The cell line HCT116 (HCT WT) was purchased from American Type Culture Collection (ATCC, Manassas, USA) and maintained in RPMI 1640 (PAN Biotech, Aidenbach, Germany) supplemented with 10% fetal bovine serum (FBS) (PAN-Biotech) and 1% Penicillin-Streptomycin (P/S) (PAN Biotech). The cell lines HCT116 p21-/- (HCT p21-/-) and HCT116 p53-/- (HCT p53-/-) were generously provided by Bert Vogelstein (Johns Hopkins). HCT p53-/- cells were maintained in McCoy’s 5A medium (Life Technologies, Darmstadt, Germany) containing 10% FBS and 1% P/S. Dubelco’s modified Eagle’s medium (DMEM) (Life Technologies) enriched with 10% FBS, 1% P/S, 1% sodium pyruvate solution (Sigma-Aldrich, Taufkirchen, Germany) and 1% MEM-Non-Essential Amino Acids Solution (Life Technologies) was used to culture HCT p21-/- cells.

The cell lines DLD-1 (DLD-1 WT) and DLD-1 CDKN1A (-/-) (DLD-1 p21-/-) were purchased from Horizon Discovery Group plc and cultivated in RPMI 1640 with 10% FBS and 1% P/S. All cell lines were cultured at 37°C in a humidified atmosphere with 5% CO_2_ and were checked routinely for mycoplasma contamination. Phase-contrast images (Magnification: 10x) were taken with the MC120 HD camera (Leica, Wetzlar, Germany) in combination with the DMi1 microscope (Leica). Images were further edited using Adobe PhotoShop CS5 and ImageJ analysis software. All cell lines were authenticated using Multiplex Cell Authetication by Multiplexion (Heidelberg, Germany). Mycoplasma free status has been verified.

**Collection of cell pellets**

Before preparing whole cell lysates, cell lines were washed on ice two times with ice cold PBS. Cells were then covered in ice cold PBS, scraped off the culture dish and transferred into 15 ml tubes (Greiner bio-one, Frickenhausen, Germany). After centrifugation (5.000 rpm, 5 min, 4°C) the supernatant was discarded, the cells were suspended in ice cold PBS and the cell solution was divided (Protein: ~60%; DNA: ~10%; RNA: ~30%) and transferred in sterile 1.5 ml tubes (Sigma-Aldrich). Centrifugation was repeated and supernatant was discarded. Cell pellets were frozen in liquid N_2_ and stored at -80 °C until further use.

**Western blotting**

A list of used antibodies can be found in the supplementary information. Cell pellets for protein analysis were thawed on ice and suspended in UREA lysis buffer (4 M UREA, 0.5% SDS, 62,5 mM Tris, pH 6.8) supplemented with 1% PMSF (Carl Roth GmbH&Co, Karlsruhe, Germany) and 1% PIC (Merck Millipore, Darmstadt, Germany). Cell lysates were stored on ice for 1 h and vortexed every 10 minutes. Lysates were then sonicated and centrifuged (14.000 rpm, 10 min, 4 °C). Protein concentration was determined using DC^TM^ Protein Assay Kit (Bio-Rad, Munich, Germany) according to supplier’s instruction and measured with VICTOR Multilabel Plate Reader (PerkinElmer, Rodgau, Germany). Protein lysates were separated by SDS-PAGE and blotted on nitrocellulose membranes overnight using Mini-PROTEAN^®^ Tetra Vertical Cell Systems (Bio-Rad) in 1x blotting buffer (25 mM Tris, 192 mM Glycin, 16% methanol, pH 8,2 – 8,4).

Anti-biotin, HRP-linked antibody (Cell Signaling Technology, Denver, USA) was used to detect the biotinylated protein ladder (Cell Signaling). Visualization of protein bands was performed using Immobilon Western Blot Chemiluminescent HRP Substrate (Merck Millipore) and detection was achieved using CoolSNAP HQ2 CCD camera (Photometrics, Tucson, AZ, USA) in combination with the Gel-Pro^®^ Analyzer Version 6.0 software (Media Cybernetics, Cambridge, UK). Furthermore, detection was also achieved using the GeneGnome XRQ Bio Imaging Chamber (Syngene Bioimaging, Cambridge, UK). Images were processed using Adobe PhotoShop CS5 and ImageJ analysis software. Band intensities were quantified using ImageJ analysis software and ratios were calculated against the GAPDH band intensity.

**RNA expression analysis**

Cell pellets for RNA analysis were thawed on ice and RNA isolation was performed using QIAzol^®^ Lysis Reagent (Qiagen, Hilden, Germany) in combination with RNeasy Mini Kit (Qiagen) according to the manufacturer’s protocols. Purified RNA was eluted in RNase free H_2_O and stored at –80 °C until further use. RNA concentration was measured using the Nanodrop^®^ ND-1000 (peqlab Biotechnologie GmbH, Erlangen, Germany). Reverse transcription was executed using Quantitect Reverse Transcription Kit (Qiagen) and RT² First Strand Kit (Qiagen) following the manufacturer’s instructions. Complementary DNA (cDNA) was stored at -20°C until further use. Amplification of cDNA was done using gene-specific primers and Quanti Tect SYBR^®^ Green PCR Kits (Qiagen) conferring the supplier’s manual.

All primers used for RT-qPCR and the gene lists for RT² Profiler PCR Arrays can be found in the Supplementary section. RT² Profiler PCR Arrays for chromatin modification enzymes (Qiagen) and for EMT (Qiagen) were performed in combination with the RT² SYBR^®^ qPCR Mastermix (Qiagen) as described in the supplier’s protocols. The data analysis was performed using the open source RT² Profiler PCR Array Data Analysis software (SABiosciences, Version 3.5). Measurement of expression values was accomplished with the CFX96^TM^ Real-Time System (Bio-Rad) and the C1000^TM^ Thermal Cycler (Bio-Rad). Expression values were normalized to human B2M or human GAPDH expression. Gene expressions are shown as the relative fold expression compared to respective control samples.

**SETD1B and Zeb1 knock-down – siRNA transfection**

For siRNA experiments, HCT p21-/- cells were seeded on 6-well cell culture plates and incubated overnight. Knockdown through transient siRNA was performed using Lipofectamine^®^ RNAiMAX reagent (Life Technologies) according to the manufacturer’s protocol. Cell lines were transfected at a confluency of 60-70% for 6 h and cell pellets were collected and stored as described before (Collection of cell pellets) at 24 and 48 h after transfection start. Protein and RNA expression analysis was performed as recently described above (Western blotting, RNA expression analysis). Final concentration of siRNAs per 6-well was 20 nM. siRNA sequences (Thermo Fischer Scientific GmbH, Erlangen, Germany). For SETD1B siRNA experiments, the following reagents were used: siSETD1B = MISSION^®^ esiRNA human SETD1B (Sigma-Aldrich, Darmstadt, Germany; #EHU137261-20UG) and siCTR = MISSION^®^ esiRNA targeting EGFP (Sigma-Aldrich, Darmstadt, Germany; #EHUEGFP-20UG). For ZEB1 siRNA experiments the following constructs were used: siCTR (5’-r(GCUACCUGUUCCAUGGCCA)d(TT)-3’; 5’-r(UGGCCAUGGAACAGGUAG C)d(TT)-3’) and siZeb1 (5’-r(AGAUGAUGAAUGCGAGUCG)d(TT)-3’; 5’- r(UGACUCGCAUUCAUCAUCU)d(TT)-3’).

**Zeb1 overexpression – Plasmid transfection**

For Zeb1 overexpression experiments HCT WT cells were seeded on 6-well cell culture plates and incubated for overnight. Transient Zeb1 overexpression through plasmid transfection was performed using Lipofectamine^®^ 3000 reagent (Life Technologies) according to the manufacturer’s protocol. Cell lines were transfected at a confluency of 60-70% over night and cell pellets were collected and stored as described before (Collection of cell pellets) at 24 and 48 h after transfection start. Protein and RNA expression analysis was performed as recently described above (Western blotting, RNA expression analysis). Final plasmid amount per 6-well was 1.5 µg.

**Immunohistochemistry – Staining protocol**

A list of the used antibodies can be found in the Supplementary Informations. Immunohistochemical (IHC) staining for haematoxylin & eosin (HE) and several antibodies were performed on formalin-fixed, paraffin-embedded (FFPE) samples. Preparation of FFPE samples was accomplished as followed: Samples were fixed in 4% formalin solution for 24 to 48 h, dehydrated in 75%, then 90% and then 100% ethanol solutions (2x for 2 h at RT for each step), cleared with 100% Xylol solution (2x for 2 h at RT) and then embedded in paraffin. FFPE sections were the prepared from paraffin blocks and used for IH staining. A detailed protocol of the IH antibody staining and the appropriate dilutions can be found in the supplementary information. Briefly, FFPE sections (2 - 4 µm) were deparaffinized, rehydrated and pretreated in a steam cooker. Bright field images (Magnification: 200x, 400x & 600x) of stained sections were taken with the Olympus XC50 camera (Olympus Corporation, Shinjuku, Japan) in combination with the Olympus BX51 microscope (Olympus Corporation) and the cellSens entry software (Olympus Corporation).

**Immunohistochemistry – Assessment of mitosis amount and vessel density**

Using a digital scanner (Panoramic-Midi and Panoramic Flash 250, 3DHISTECH Ltd., Budapest, Hungary) the HE stained FFPE sections were digitized to evaluate the mitosis amount and the vessel density of each sample. To assess the amount of mitoses, 5 high power fields (HPF; Magnification: 400x) were analyzed by an experienced pathologist and the numbers of mitoses in each HPF were documented. The mean of the 5 HPF values was calculated to generate the mitoses value of each HE and then the mean values (mitoses) of all sections from one cell line (HCT WT and HCT p21-/-) were calculated and compared between both cell lines. The assessment of the vessel density was performed using the CaseViewer software (Panoramic-Midi and Panoramic Flash 250, 3D-Histech) using the digitized HE stained FFPE sections. First, the tumor cell mass was marked, and the tumor cell area was measured. Next, the area of infiltrated vessels was measured. Vessel density was calculated as a ratio of the sum of vessel by the sum of tumor cell area for each slide. The mean values (vessel density) of all sections from one cell line (HCT WT and HCT p21-/-) were calculated and compared between both cell lines.

**Chromatin-immunoprecipitation (ChIP)**

A list of the used antibodies can be found in the supplementary information. Briefly, HCT cell lines were seeded on 15 cm cell culture dishes and incubated for ~ 72 h. Cells were collected and ChIP was performed using the ChIP-IT High Sensitivity^®^ kit (Active Motif, La Hulpe, Belgium) according to the supplier’s instructions. For one ChIP reaction, 30 µg of chromatin were used and following gene regions (in relation to transcription start sites) were amplified and analyzed by qPCR as previously described (RNA expression analysis): SETD1B (-52 bp to +56 bp), VIMENTIN (-459 bp to -298 bp) and ZEB1 (-323 bp to -147 bp). The regions SETD1B.NEG (+915 bp to +1151 bp), VIMENTIN.NEG (-1.971 bp to -1.824 bp) and ZEB1.NEG (-1953 bp to -1872 bp) were used as negative controls. LAMC2 (-174 bp to -25 bp) served as a positive control for the ZEB1-ChIP. Fold enrichment values were calculated against the IgG control as previously described^15^.

**Immunofluorescence**

HCT cell lines were grown on glass cover slips, fixed in 3% paraformaldehyde solution (Sigma) for 15 min at RT and washed several times with PBS. After permeabilization with 0.2% Triton X-100 (Sigma) for 5-10 min at RT and washing with PBS, cells were incubated in blocking buffer (1% BSA in PBS) for 10 min at RT and washed again with PBS. Immunofluorescence staining of f-Actin filaments was performed with Alexa Fluor^®^ 488 Phalloidin reagent (Life Technologies) for 30 min at RT and cells were mounted on object slides with ProLong^®^ Gold Antifade reagent (Life Technologies) according to manufacturer’s guidance. Confocal images were acquired using Laser Scanning Microscopy system LSMT-PMT Observer Z1 (Carl Zeiss AG, Oberkochen, Germany), the ZEN imaging software (Carl Zeiss AG, Oberkochen, Germany) and a 63x oil objective. Images were edited using ZEN imaging software, Adobe PhotoShop CS5 and ImageJ analysis software.

**Chorioallantoic membrane (CAM) assay**

Fertilized and specific pathogen-free eggs (VALO BioMedia) were used for the *in* vivo CAM assay. At day 8 of embryonic development the eggs were opened on the flattened pole and the egg shell membrane was removed. The window was covered with sterile tape and the eggs were further incubated. On day 9, HCT cell lines were resuspended in a Matrigel/medium mixture (1:1) (Corning Incorporated, NY, USA). Drops of Matrigel/cell solution were pipetted onto sterile culture dishes. One Matrigel/cell pellet per egg was carefully placed on top of the CAM. Eggs were further incubated for 5 days. For IHC staining and size measurement the microtumors were pre-fixed with 4% phosphate-buffered formalin solution and then carefully removed using sterile scissors and tweezers and transferred to PBS-containing culture dishes. Pre-fixed samples were placed on pieces of filter paper, photos (Magnification: 10x) of the harvested tumors were taken using a USB microscope (Traveler) in combination with the CamApp software (AVEO Corporation, MA, USA) and sample dimensions (height, length, width) were measured using a ruler.

Sample volume was calculated using following equation: V = (π/6)*(h*l*w). Samples were then further incubated at RT in 4% formalin solution for ~ 24 to 48 h and processed as previously described (Immunohistochemistry) to create FFPE blocks. HE and IHC stainings of FFPE sections were performed as previously mentioned (Supplements: Immunohistochemistry – Staining protocol). Based on HE staining, 5 randomly picked high power fields (Magnification: 400x) of each sample were used to determine the amount of mitoses and to analyze morphological cell features. Protein and RNA fragments were collected from un-fixed samples. Samples were therefore removed directly from the egg, shortly washed in PBS and cut into two pieces per sample (Protein: ~65 %, RNA: ~35%). Fragments were then transferred to sterile 1.5 ml tubes, frozen in liquid N_2_ and stored at -80 °C until further use. Protein and RNA analysis was performed as described above (Western blotting, RNA expression analysis).

**Bisulfite conversion and pyrosequencing analysis**

Isolation of genomic DNA was performed using NucleoSpin^®^ Tissue kit (Macherey-Nagel) following the manufacturer’s instructions and bisulfite conversion was accomplished with the CpGenome Universal DNA Modification kit (Chemicon) according to the supplier’s manual. Amplification of genomic DNA was done using Multiplex PCR Kit (Qiagen) in accordance with the company’s protocol and methylation status of VIMENTIN and ZEB1 promoter regions (relative to TSS) was analyzed using PyroMark^®^ Q24 reagents (Qiagen) and PyroMark Q24 system (Qiagen) as suggested by the company.

**Structural modelling of protein-protein interactions**

Modelling of Zeb1:

Since no crystallographic or NMR structures of Zeb1 are available in the PDB, Zeb1 was modelled using a combination of homology modelling and structure prediction approaches as mentioned above. PDB ids 2WBT,2YTR_A, 2RSJ_A, 2E19, 2COT_A and 1A1I were used for homology modelling and structure prediction was done for the remaining regions using ITASSER^18^.

Modelling of SETD1B:

Regions representing the RRM domain of SETD1B and the SET Post SET Domains regions were modelled using homology modelling tool MODELLER^17^ using the templates 3S8S and 5F6K respectively.

Modelling of the (Zeb1-SETD1B) complexes

The complex of (Zeb1-SETD1B) was formed by docking the individual proteins using the Protein-Protein docking tool ClusPro^19,20^. The complexes with the least energy were chosen for the analysis of interactions between the proteins considered. The cluster scores of the complexes from the ClusPro server were used to understand the energy profiles of the (Zeb1-SETD1B) complex. The ionic, hydrophobic, hydrogen bond interactions were identified and analyzed using the Protein Interaction Calculator (PIC)^21^. All renderings were done using CHIMERA^22^.

**Bioinformatic workflow to determine genes potentially regulated by ZEB1 and p21**

For the identification of p21-dependent genes, Pearson correlation dissimilarity measure was used to calculate distance between HCT116 WT and HCT116 p53-/- cell lines. Similar gene expression values in HCT116 WT and HCT116 p53-/- cell lines led to overlapping clusters. On the other hand, the cell line HCT116 p21-/- samples were separated. In order to identify potential ZEB1 targets, genome wide search for ZEB1 E-Box transcription factor binding sites (5'-CANNTG-3') was performed using TRANSFAC^®^ database (BIOBASE GmbH, Wolfenbüttel, Germany; Version 2015.3). Furthermore, TRANSFAC^®^ database was used to identify ZEB1 Z-Box transcription factor binding sites (5’- CAGGTG- 3’ or 5’- CAGGTA-3’) in 15 p21 chromatin modifiers. Using experimentally validated and predicted binding sites for each human gene using a combination of Text Mining (large scale search of biological published papers and other public resources) and experimentally validated data (ChIP-seq data) were used. To identify genes potentially regulated by both, p21 and ZEB1, the statistical analysis of the PCR Array Dataset and the genome wide search for ZEB1 transcription factor binding sites using TRANSFAC^®^ software were combined.

**Statistical analyses**

Statistical analyses for the following experiments were performed using Microsoft Excel 2010 software (Microsoft Corporation, WA, USA) and GraphPad Prism 7 software: ChIP, mitosis amount, RT-qPCR, tumor size and western blot. Each experiment represents two or more biological replicates and the data are depicted as mean±s.d. or mean±s.e.m. All experiments were technically repeated at least two times in the laboratory. Unpaired two-tailed Student’s *t*-test was applied to compare groups of independent samples. Statistical analyses of clinical data sets were performed using GraphPad Prism 7 software (GraphPad Software, Inc.) and SPSS Version 21 (IBM). Following clinical data sets were used: GSE13067, GSE13294, GSE14333, GSE17536, GSE20916, GSE2109, GSE33113, GSE35896 and GSE42284.

Comparison of gene expression differences between two groups of CMS cohorts was accomplished by Mann-Whitney *U*-test. Correlations of gene-gene pairs of the gene set of CMS cohorts were examined calculating the Pearson correlation coefficient. Log rank test to compare gene expression profiles and stratified patients based on death information was performed to test significance in survival analyses with Kaplan-Meier curves for the GSE17536 data set from Gene Expression Omnibus. Death from unrelated causes has been censored.
