# Supplementary material for: EMT transcription factor ZEB1 alters the epigenetic landscape of colorectal cancer cells
Source: Cell Death Dis. 2020 Feb 24;11(2):147. doi: 10.1038/s41419-020-2340-4 (PMC7040187; doi:10.1038/s41419-020-2340-4)
Supplement: Supplementary file 6 — Suppl. Tables [file 41419_2020_2340_MOESM6_ESM.docx]

**Supplemental Information – Tables**

**Supplementary Table 1:**

**List of primers used in RT-qPCR experiments**

**Supplementary Table 2:**

**List of primers used in ChIP experiments**

**Supplementary Table 3:**

**List of primers used in pyrosequencing experiments**

**Supplementary Table 4:**

**Antibodies used in western blot experiments**

**Supplementary Table 5:**

**Antibodies used for immunohistiochemistry experiments**

**Supplementary Table 6:**

**Antibodies used for chromatin immunoprecipitation experiments**

**Supplementary Table 7:**

**PCR run protocols**
